# *In vivo*^99m^Tc-HYNIC-annexin V imaging of early tumor apoptosis in mice after single dose irradiation

**DOI:** 10.1186/1756-9966-28-136

**Published:** 2009-10-08

**Authors:** Ming-fang Guo, Yaqing Zhao, Rong Tian, Lin Li, Leiming Guo, Feng Xu, Yong-mei Liu, Yong-bo He, Sen Bai, Jin Wang

**Affiliations:** 1 Division of Abdominal Tumor, Cancer center, West China Hospital, Sichuan University, Chengdu, Sichuan, PR China; 2 Department of Nuclear Medicine, West China Hospital, Sichuan University, Chengdu, Sichuan, PR China; 3 Division of Radiation Physics center, Cancer center, West China Hospital, Sichuan University, Chengdu, Sichuan, PR China; 4 Division of Thoracic Tumor, Cancer center, West China Hospital, Sichuan University, Chengdu, Sichuan, PR China

## Abstract

**Background:**

Apoptosis is a major mode of hematological tumor death after radiation. Early detection of apoptosis may be beneficial for cancer adaptive treatment. ^99m^Tc-HYNIC-annexinV has been reported as a promising agent for in vivo apoptosis imaging. The purpose of this study is to evaluate the feasibility of in vivo^99m^Tc-HYNIC-annexinV imaging of radiation- induced apoptosis, and to investigate its correlation with radiosensitivity.

**Methods:**

Ten days after inoculation of tumor cells in the right upper limbs, the mice were randomly divided into two groups. The imaging group (4 mice each level, 4 dose levels) was injected with 4-8 MBq ^99m^Tc-HYNIC-annexinV 24 hours after irradiation and imaged 1 hr post-injection, and the mice were sacrificed immediately after imaging for biodistribution analysis of annexin V. The observation group (4 mice each level, 2 dose levels) was only observed for tumor regression post-radiation. The number of apoptotic cells in a tumor was estimated with TUNEL assay.

**Results:**

The ^99m^Tc-HYNIC-annexin V uptake in E14 lymphoma significantly increased as the radiation dose escalated from 0 to 8 Gy, and significantly correlated with the number of TUNEL-positive cells (r = 0.892, P < 0.001). The Annexin-V uptake and the number of TUNEL-positive cells in El4 lymphoma were significantly greater than those in S180 sarcoma. With 8 Gy, S180 sarcoma tumor showed scanty apoptosis and less shrinkage while El4 lymphoma showed remarkable apoptosis and complete remission.

**Conclusion:**

^99^mTc-HYNIC-annexinV in vivo imaging is a feasible method to detect early radiation-induced apoptosis in different tumors, and might be predictive for radiation sensitivity.

## Background

Apoptosis is a major mode of hematological tumor death after ionizing irradiation and is closely correlated with tumor sensitivity to radiation. The radiation induced apoptosis can be classified as pre- and post-mitotic based on the onset time [1,2]. Detecting the early phase of radiation-induced apoptosis is of special value for the prediction of response to a certain treatment as well as for early intervention with individualized treatment strategies. At the early stage of apoptosis, the membrane-bound lipid phosphatidylserine (PS), which is normally restricted to the inner leaflet of the plasma membrane lipid bilayer by an adenosine triphosphate-dependent translocase, becomes exposed at the outer leaflet of the plasma membrane bilayer [3]. Annexin V is an endogenous human protein and has a high affinity for membrane-bound PS. The number of annexin V binding sites per cell with the onset of apoptosis increases 100-to 1,000-fold during apoptosis. PS exposure on the cell surface closely follows caspase-3 activation and occurs well before DNA fragmentation. Therefore annexin V is a sensitive marker of the early to intermediate phases of apoptosis. Human annexin V can be conjugated with hydrazinonicotinamide (HYNIC) and radio-labeled with ^99m^Tc to get ^99m^Tc-HYNIC-annexin V, with a high labelling rate and radiochemical purity and stability [4]. The In vivo ^99m^Tc-HYNIC-annexinV apoptosis imaging has been reported to be able to predict the severity of myocardium infarction, organ transplantation rejection and response to tumor chemotherapy treatment [5,6]. Encouraging results were reported by some pilot studies [7,8] that early phase ^99m^Tc-HYNIC-annexin V scintigraphy (TAVS) after radiotherapy in patients may be useful as a predictive test for treatment response. However, the potential value of ^99m^Tc-HYNIC-annexin V imaging in the evaluation of radiation-induced apoptosis has yet to be established.

In order to evaluate the value of ^99m^Tc-HYNIC-annexin V imaging in detecting early phase apoptosis in tumors after single dose irradiation and in predicting tumor response to radiotherapy, a radiation murine tumor model was established, and the relevance of TAVS image to apoptosis and radiation sensitivity was explored.

## Methods

### Animals

Male C57BL/6 mice and Kunming mice were obtained from the breeding facility of the Experimental Animal Center, West China Medical Center, Sichuan University. All mice were used between 6 and 12 weeks of age, and weighed 18 to 22 g. Care of all experimental animals was in accordance with institutional guidelines and approved protocols.

### Cell Culture Technique

The C57BL/6 mice derived EL4 lymphoma cell line was obtained from the Transplantation Immunology Laboratory of West China Hospital, Sichuan University. The Kunming mice derived S180 sarcoma cell line was obtained from the Tumor Biotherapy Laboratory of West China Hospital, Sichuan University. Both EL4 and S180 cell lines were grown as cell suspensions in RPMI 1640 medium, supplemented with 10% (v/v) fetal bovine serum and 290 μg/mL L-glutamine, 100 U/mL penicillin and 100 μg/mL streptomycin. Cells were maintained in the logarithmic growth phase at a concentration of 1-5 × 10^5 ^cells/mL at 37°C in a 5% CO_2 _in air atmosphere under aseptic conditions.

### Flow cytometry (FCM) assessment of apoptosis

Groups of EL4 lymphoma cells in logarithmic growth phase were irradiated with a single dose of: 0 Gy, 2 Gy, 4 Gy or 8 Gy; the S180 sarcoma cells received only 0 Gy or 8 Gy. The 0 Gy group was served as the unirradiated control for both tumors. Irradiation was with 4 MV X-rays generated by the Elekta Precise linear accelerator (Elekta, Sweden) using 100 cm SSD,10 cm × 10 cm portal size, with the cell culture flask lying on a 1.0 cm thick Perspex. Twenty-four hours after irradiation, the samples were harvested and stained with Annexin V-FITC and PI for 15 min at 25°C by using a commercial kit (BD Pharmingen, USA). Cells were washed twice with PBS and re-suspended in buffer solution (1 × 10^6 ^cells per ml). Stained cells were analyzed with a flow cytometer (BD, FACSAria™) within 1 hour of staining, as described in the manufacturer's manual.

### Mouse bearing tumor model

The EL4 and S180 cells in logarithmic phase were harvested and washed three times with PBS, and re-suspended in serum-free RPMI 1640 medium at a concentration of 1.75 × 10^7^cells per ml. A volume of 0.2 ml (3.5 × 10^6^cells) tumor cell suspension was injected subcutaneously ventral to the right axilla of the mice (C57BL/6 for EL4, Kunming mice for S180). Mice were monitored for tumor burden by measuring the tumor size daily using a vernier calliper. Irradiation began when the tumor diameter attained 1.0 cm.

### Preparation of ^99m^Tc-HYNIC-Annexin V

Human annexin V freeze-dried powder was purchased from Beijing Huada Protein Development Center Co. Ltd (Beijing, China). Human annexin V was conjugated with hydrazinonicotinamide (HYNIC), using methods described by Blankenberg et al. [5]. Derivatized HYNIC-annexin V was radio-labelled with a ^99m^Tc tricine precursor complex according to literature methods [5,9-11]. After chelating with the ^99m^Tc tricine precursor complex, the radio-labeling efficiency was measured by using thin-layer chromatography Silica Gel (TLC-SG), with methyl ethyl ketone and normal saline as the developing solvent. The radiochemical purity of the tracer product was then measured with High Performance Liquid Chromatography. The radio-labelled material, prepared as described above, was diluted to have specific activities ranging from 400-800 MBq μg^-1 ^1 ml^-1 ^which was ready for use.

### Tumor irradiation

The tumor-bearing mice were randomly divided into an imaging group which was irradiated and imaged using ^99m^Tc-HYNIC-Annexin V, and an observation group which was only observed for tumor regression after single-dose irradiation. The EL4 lymphoma imaging group was subdivided into 4 single-dose levels: 0, 2, 4, and 8 Gy, while the S180 sarcoma imaging group received only 2 dose levels (0 and 8 Gy), with 4 mice each level. The observation only groups of EL4 lymphoma and S180 sarcoma both received the same dose levels of 0 Gy or 8 Gy (4 mice each level). The tumors were irradiated with the 4 MV X-rays (SSD 100 cm, 1.5 cm × 1.5 cm portal) with a 0.5 cm thick tissue-equivalent material applied to the tumor surface. The mice were anesthetized before irradiation by intraperitoneal injection of 0.15 ml of 0.7% pentobarbital and immobilized with tapes. Experiments were repeated three times.

### ^99m^Tc-HYNIC-annexin V imaging of radiation-induced apoptosis

At 24 hours after radiation, 0.2 ml (4-8 MBq) of the prepared ^99m^Tc-HYNIC-annexinV was injected into each mouse in the imaging groups through the tail vein. Planar images were obtained 2 hours later, using a single-head γ camera (Meridian Philips Medical Systems) equipped with a parallel-hole collimator. The energy window was centered at 140 keV with a window width of 20%, and the matrix was to 256 × 256 with a magnification factor of 3.0. The acquisition time was 1 min/image. The tumor size of mice in the observation groups was measured daily after irradiation.

### Biodistribution of ^99m^Tc-HYNIC-annexin V in tumors

Blood was drawn from the post-glomus venous plexus immediately after ^99m^Tc-HYNIC annexin V imaging and mice were decapitated afterwards. The blood (B), the tumor (T), and muscle (M) were excised from the mice and weighed and then counted in a well-type γ Counter (Xian Manufacture, China) for the evaluation of ^99m^Tc-annexin V biodistribution (energy peak at 140 keV and 10 s). The percentage of injected dose per gram of tissue (%ID/g) was calculated. The T/M and T/B ratio were calculated for correction of background radio-activity and decay of ^99m^Tc-HYNIC annexin V tracer.

### Histocytochemical study of apoptosis in tumor tissue

Tumor apoptosis was assessed by in situ end-labelling of DNA fragments (TdT-mediated dUTP-biotin nick end labelling, TUNEL) using a commercially available kit (Roche Applied Science). The fresh tumor tissue was fixed in 10% formaldehyde for 24 hours, dehydrated, paraffin-embedded and cut into 5- μm thick sections which were subsequently mounted on slides, rehydrated before stained with TUNEL for microscopic analysis. The mean number of apoptotic cells was counted in 10 randomly selected high-power fields.

### Statistical analyses

Data were analyzed using the SPSS 13.0 software package. All variables were expressed as mean (M) and standard deviation (SD). All statistical comparisons of mean values were performed with the F test (one-way ANOVA). Linear correlation coefficients were calculated using a least squares linear regression analysis. A significance level of *P *< 0.05 was considered significant.

## Results

### Effect of different radiation doses on apoptosis in EL4 cells

The EL4 cells were irradiated in single-dose of 0, 2, 4 and 8 Gy groups, respectively. After irradiation, the cells were maintained in suspension culture for 24 hours, and then analyzed with FCM. As shown in Table 1, the EL4 cells had spontaneous apoptosis even when no radiation was given (0 Gy), and the number of apoptotic cells increased as radiation dose was escalated from 2 to 8 Gy.

**Table 1:** The change of apoptotic rate in EL4 lymphoma cells evaluated by FCM after different doses of 4 MV X-ray radiation

**Dose(Gy)**	Apoptotic rate* (%)
0	3.13 ± 0.42
2	6.80 ± 0.20
4	12.60 ± 0.56
8	16.17 ± 0.85

*Value is expressed as Mean ± SD.

**Figure 1:**
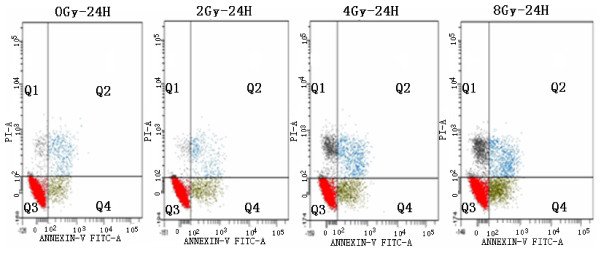
**Flow cytometry results for El4 lymphoma cells 24 hours after single-dose irradiation**. Using Annexin V-FITC and PI stain, it showed that the ratio of apoptotic cells increased with the escalation of dose. The abscissa represents the number of AnnexinV positive cells; the ordinate represents the number of PI positive cells. Q1 represents the necrotic cell potion, Q2: apoptotic cells; Q3: normal cells; Q4: early phase apoptotic cells.

The apoptotic cell fractions (measured by FCM based on Annexin V-FITC and propidium iodide (PI) staining) of EL4 cells that received different single-irradiation doses (0 - 8 Gy) are shown in Figure 1. It shows that the number of necrotic (Q1) and apoptotic cells (Q2+Q4, Q4 represents the early phases of apoptosis) both significantly increased as the radiation increased from 0 to 8 Gy.

### In vivo ^99m^Tc-HYNIC-annexin V imaging

**Figure 2:**
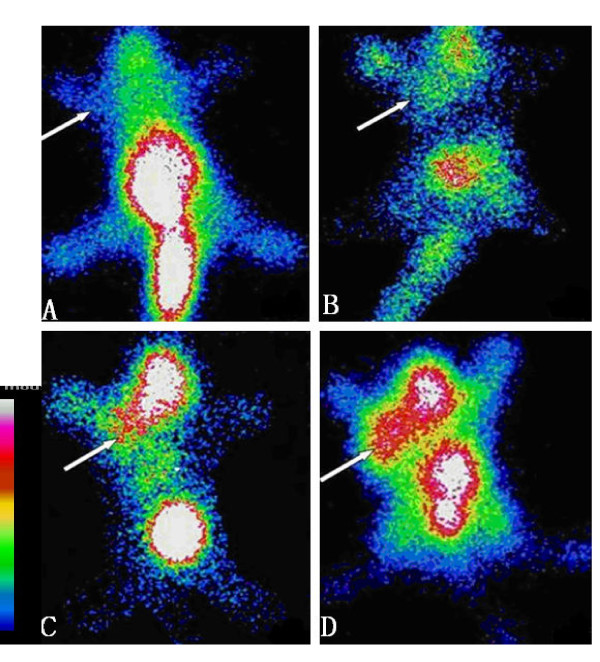
**Representative ^99m^Tc-HYNIC-annexin V scintigraphy (TAVS) images of EL4 lymphoma bearing mice treated with irradiation**. Mice were injected 4-8 MBq radiolabeled annexin V 24 hours post-radiation and imaged 2 h later. The images show increased annexin V uptake in tumor as radiation dose increased. The white arrow indicates the implanted tumor. A: 0 Gy; B:2 Gy; C:4 Gy; D:8 Gy.

**Figure 3:**
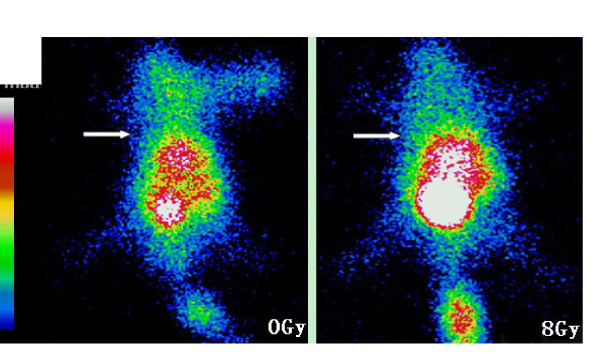
**Annexin V imaging of S180 sarcoma bearing mice treated with irradiation**. The images show insignificant annexin V uptake in tumor with radiation dose of 8 Gy comparing to 0 Gy control. The white arrow indicates the implanted tumor. A: 0 Gy; B:8 Gy.

The labelling efficiency of the HYNIC-annexin V sample was 90%, with a radiochemical purity of 95%. Two hours after injection of 0.2 ml of the prepared 99mTc-HYNIC annexin (4-8 MBq), whole body planar imaging was performed on tumor bearing mice which had received different single-doses of radiation. As shown in Figures 2 and 3, without radiation (0 Gy), the radioactivity uptake in EL4 lymphoma and S180 sarcoma was similar to that of the background; the tumors were not clearly shown in ^99m^Tc-HYNIC-annexinV imaging. Moreover, the images in control animals (0 Gy) demonstrated a high concentration of radio-labelled annexin V in the heart and bladder, with a lesser distribution in other organs (Figures 2A and 3A). The tracer uptake shows accumulation in the head and neck and thymus region in EL4 lymphoma irradiated with 4 Gy and 8 Gy (Figures 2C and 2D). The increased density of tracer in the tail (Figures 2A and 3B) was due to the tracer at the site of injection. The liver and kidneys were not visualized as separate structures. It demonstrated (Figures 2B to 2D) that for EL4 lymphoma, as the radiation dose was escalated from 2 to 4 and 8 Gy, there was a marked increase in tumor uptake of ^99m^Tc-HYNIC annexin V. The irradiated tumor image became clearer. However, in S180 sarcoma bearing mice, even with 8 Gy irradiation, the tumor uptake of ^99m^Tc-HYNIC- annexin V was similar to that of the background; and the tumor was not clearly shown in imaging. The ^99m^Tc-HYNIC- annexin V uptake concentration was high in bladder, liver and kidney.

### Biodistribution of ^99m^Tc-HYNIC- annexin V and tumor apoptosis after irradiation

The control and irradiated mice were sacrificed immediately after ^99m^Tc-HYNIC-annexin V imaging. Biodistribution assays were performed with a well-type γ-counter. The radioactivity parameters measured (T/M and T/B ratios) are shown in Tables 2 and 3.

**Table 2:** Biodistribution of ^99m^Tc-HYNIC-Annexin-V in EL4 lymphoma and the number of apoptotic cells after single-dose irradiations

		**Dose**	**(Gy)**			***p***	
	
	**0**	**2**	**4**	**8**	**0 vs.2**	**2 vs.4**	**4 vs.8**
%ID/g	0.160 ± 0.013	0.272 ± 0.021	0.312 ± 0.020	0.355 ± 0.025	<0.001	0.017	0.009
T/B	0.729 ± 0.037	1.252 ± 0.086	1.396 ± 0.021	1.661 ± 0.072	<0.001	0.005	<0.001
T/M	2.575 ± 0.154	4.522 ± 0.554	5.191 ± 0.511	7.138 ± 0.266	<0.001	0.039	<0.001
Apoptotic cells	1.405 ± 0.191	2.459 ± 0.370	4.364 ± 0.778	6.953 ± 0.673	0.002	0.004	0.002

Results expressed as mean ± SD. %ID/g = percentage injected dose per gram of tumor tissue; T: ^99m^Tc-HYNIC annexin-V uptake in tumor; B: ^99m^Tc-HYNIC-annexin V uptake in blood; M: ^99m^Tc-HYNIC annexin-V uptake in muscle. Apoptotic cells were counted as the number of TUNEL positive cells per mm^2 ^of each examined section.

**Table 3:** Biodistribution of ^99m^Tc-HYNIC-Annexin-V in S180 sarcoma and the number of apoptotic cells after single-dose irradiations

	**Dose**	**(Gy)**	
		
	**0**	**8**	***p***
%ID/g	0.097 ± 0.008	0.102 ± 0.008	0.464
T/B	0.475 ± 0.019	0.465 ± 0.031	0.608
T/M	1.241 ± 0.046	1.501 ± 0.167	0.024
Apoptotic cells	0.740 ± 0.362	1.627 ± 0.121	0.004

The abbreviations: the same as in Table 2.

At 0 Gy (control), the percentage injected dose per gram of tissue (%ID/g) in the tumor was low, with the T/B value of (0.7294 ± 0.0365) for EL4 lymphoma and (0.4748 ± 0.0194) for S180 sarcoma, implying less uptake of tracer in tumor than in the blood when unirradiated. However, the T/M value was (2.5745 ± 0.1538) for EL4 lymphoma and (1.2412 ± 0.0463) for S180 sarcoma, suggesting greater tracer uptake in tumor than in muscle. It could also be observed that the level of ^99m^Tc-HYNIC-annexin V uptake in control (0 Gy) tumor was much lower for S180 sarcoma than for EL4 lymphoma, implying lower spontaneous apoptosis in S180 sarcoma tumor compared to EL4 lymphoma.

Compared to the unirradiated control, the %ID/g in the irradiated EL4 lymphoma increased 1.7 to 2.3 fold, the T/B increased 1.7 to 2.3 fold, and T/M increased 2.0 to 2.8 fold, indicating increased uptake of ^99m^Tc-HYNIC- annexin V with irradiation and the increment was dose dependent. As shown in Table 2, in EL4 lymphoma, the uptake of ^99m^Tc-HYNIC-annexin V significantly increased as radiation dose rose from 0 to 8 Gy (*P *< 0.05).

On the contrary, in S180 sarcoma bearing mice, compared to the 0 Gy control, the %ID/g, T/B and T/M with 8 Gy irradiation only increased slightly (Table 3), indicating a low level of apoptosis in S180 cells after radiation. For S180 sarcoma, there were no significant differences in %ID/g and T/B ratio between the 0 Gy and 8 Gy groups (*P *> 0.05), but the T/M ratio in the 8 Gy group was significantly higher than that of the 0 Gy group (*P *= 0.024), suggesting higher uptake of tracer in blood but low level in muscle.

Comparing the radioactivity distribution in tumor between EL4 lymphoma and S180 sarcoma bearing mice, it was shown that for the same radiation dose (0 Gy and 8 Gy), the %ID/g, T/B and T/M of EL4 lymphoma were significantly higher than those of the S180 sarcoma group (both *P *< 0.001).

### Correlation between apoptotic cell number and tracer uptake in tumor

**Figure 4:**
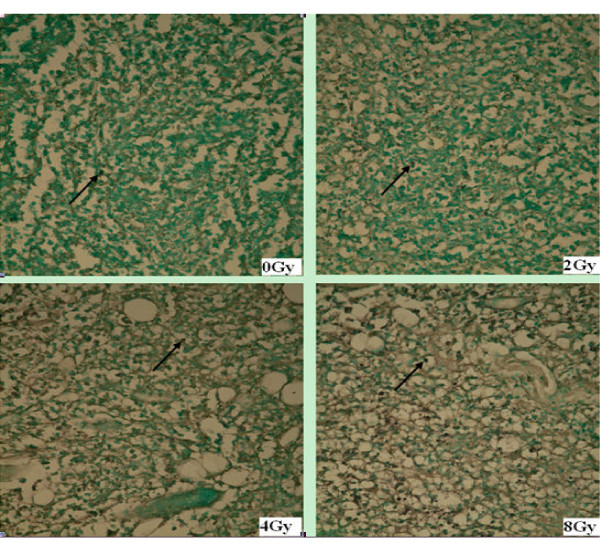
**TUNEL assay for EL4 transplant lymphoma after irradiation**. In pathological sections (× 100) of EL4 transplant lymphoma after single-dose irradiation, the black arrow indicates the TUNEL positive apoptotic cells. It shows that the number of apoptotic cells increase as the radiation dose is escalated from 0 to 8 Gy.

**Figure 5:**
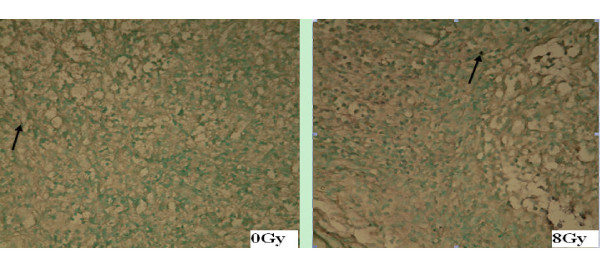
**TUNEL assay for S180 transplant sarcoma after irradiation**. In pathological sections of S180 sarcoma after irradiation (× 100), the black arrow indicates the TUNEL positive apoptotic cells. It shows that the number of apoptotic cells increases as radiation of 8 Gy is delivered comparing to that of the 0 Gy control.

The paraffin embedded tumor samples were stained for apoptosis by TUNEL and studied under a light microscope after biodistribution assay. TUNEL staining positive cells demonstrated brown staining of the tumor cell nuclei (Figures 4 and 5).

The degree of tracer uptake in tumor correlated well with the apoptotic rate evaluated by TUNEL assay. In EL4 lymphoma, the apoptotic rate significantly increased as the dose increased from 2 to 8 Gy (Table 2). In S180 sarcoma, the apoptotic rate measured by TUNEL assay was significantly higher in the 8 Gy group than that in 0 Gy group (Table 3). Similar to the biodistribution results, the corresponding apoptotic rate measured by TUNEL in the EL4 lymphoma was also significantly higher than that of the S180 sarcoma for both 0 Gy (*P *= 0.017) and 8 Gy (*P *< 0.001). The increment of apoptotic cells at 8 Gy relative to 0 Gy was less in S180 sarcoma than that in the EL4 lymphoma, which agrees well with the TAVS imaging results.

**Figure 6:**
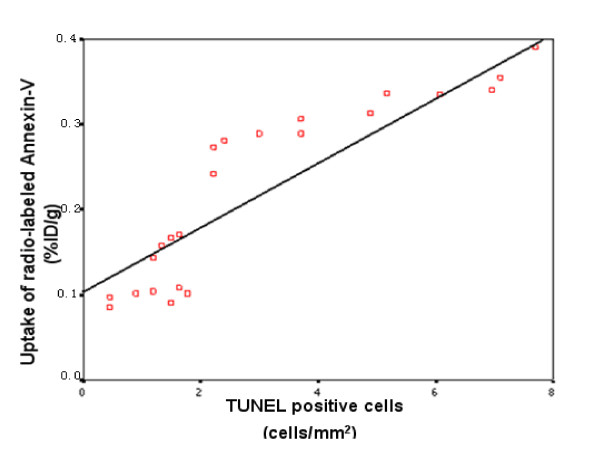
**Correlation of TUNEL positive cells and ^99m^Tc-HYNIC-annexin V uptake in EL4 and S180 tumors**. The plot shows the number of apoptotic cells (TUNEL positive) is linearly correlated with the uptake of the radio-labeled Annexin-V in the murine transplant tumors, showing that the Annexin-V imaging may illustrate different degrees of radiation induced apoptosis.

As shown in Figure 6, when data from all tumor samples were combined (EL4 and S180 tumors were not distinguished from each other), it could be observed that the number of apoptotic cells (abscissa) was linearly correlated with the percentage of ^99m^Tc-HYNIC- annexin V taken up by all tumors (ordinate), with a correlation coefficient (r) of 0.892 and a corresponding *P *value of < 0.001. These results indicated that the degree of radiation induced apoptosis in tumor could be represented by the ^99m^Tc-HYNIC- annexin V activity taken up in EL4 and S180 tumors. However, there are systematic deviations of points from the line, e.g., a sigmoid between 0.08 and 0.28 on the ordinate followed by a more gradual linear increase between 2.8 and 4.

### Tumor regression after irradiation

**Figure 7:**
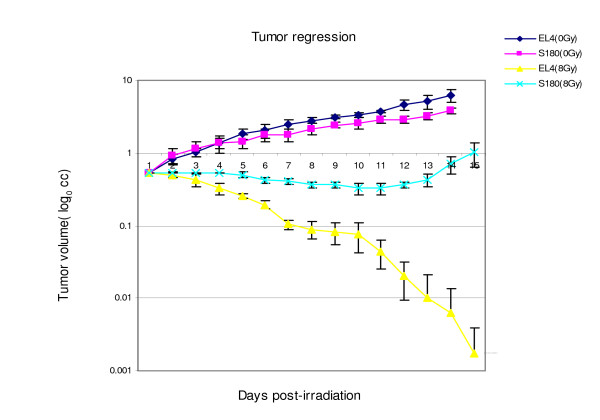
**Semilog plot of tumor regression in EL4 and S180 tumors after 8 Gy single-dose irradiation**. The tumor volume (cc) in logarithmic scale (ordinate) is plotted against days (abscissa) after radiation. The unirradiated EL4 (EL4 0 Gy) and S180 (S180 0 Gy) controls show exponential growth. EL4 lymphoma is more radiation sensitive with a complete regression, while S180 sarcoma is less radio-sensitive which slightly shrank after radiation and relapsed at 13th day.

To evaluate the tumor response to radiation, the regression of EL4 lymphoma and S180 sarcoma in mice after single-dose irradiation with 8 Gy was observed (Figure 7). Without irradiation (0 Gy), the EL4 lymphoma grew with a daily increment of 0.1 cm in diameter and reached 5.1 cc (SD = 1.1) 13 days after tumor inoculation in mice. After a single 8 Gy irradiation, the EL4 lymphoma began to shrink on the second day and the tumor underwent significant necrosis on the 6^th ^day after irradiation and disappeared completely on day 13.

For S180 sarcoma, without irradiation, the mean tumor volume grew to 3.2 cc (SD = 0.3) 13 days after inoculation of tumor in mice. After a single 8 Gy irradiation, S180 sarcoma mean volume showed minimal regression to 0.32 cc (SD = 0.06) on day 12. The S180 tumor re-grew and reached the pre-irradiation size on the 13^th ^day after irradiation, suggesting loss of tumor control. The results implied that with same dose irradiation, the EL4 lymphoma is more radiation-sensitive than S180 sarcoma.

## Discussion

In this study, ^99m^Tc-HYNIC-annexin V was conjugated and radio-labelled, and successfully applied to image the radiation-induced apoptosis in the murine tumor model. The in vivo and in vitro dose response relationships of radiation- induced apoptosis were analyzed. The in vivo apoptosis imaging was compared between two tumors with different radiation responsiveness.

The ^99m^Tc-HYNIC-annexin V imaging showed that the physiologic uptake of ^99m^Tc-HYNIC-Annexin V was mainly in the heart, kidneys, bladder, liver and spleen. The accumulation of the tracer in the head and neck and thymus in EL4 lymphoma-bearing mice at 4 and 8 Gy was significant. This was assumed to be due to increased radiation scatter to the tissues near the tumor providing greater radiation doses, thus resulting in increased apoptosis. Our results are consistent with those described in the literature, in which the tracer density in the thymus of an EL4 thymoma murine model was also elevated [12]. However, the high tracer uptake in head and neck or thymus was not observed in the Kunming mice bearing S180 sarcoma, indicating different normal tissue responses of two mouse strains.

Our results showed that at 24 hours, ^99m^Tc-HYNIC-annexin V imaging can show clearly the early phase apoptosis after single-dose irradiation. In this study, TUNEL staining was chosen to measure apoptosis rate, following the successful reports on its predictive value for apoptosis from other studies [[5,7,11], and [12]]. In both EL4 and S180 tumors, the number of apoptotic cells measured by TUNEL assay was positively correlated with the uptake of radio-labeled annexin V (Figure 6), suggesting that the application of ^99m^Tc-HYNIC-annexin V to evaluate early-phase radiation-induced apoptosis is feasible. The observation is consistent with the literature report that externalization of PS in cell membrane might appear as early as 1 to 5 hours after injury stimulation, but only the PS externalization at 9 to 24 hours was related to apoptosis [13]. Moreover, if detected later, the sensitivity of PS to predict apoptosis would be reduced due to the clearance of PS expression cells by macrophages [14,15]. Our results agree well with those of a reported study [12] that also correlated TUNEL assay with ^99m^Tc-HYNIC-annexin V uptake in a murine thymoma model to evaluate tumor response after radiation or cytotoxic drug treatment. It was postulated that ^99m^Tc-HYNIC-Annexin V may be an ideal agent for imaging of early apoptosis in response to treatment. Mochizuki et al. [11] has similarly found in a KDH-8 liver cancer murine model that annexin V imaging could accurately image the cyclophosphamide induced early apoptosis. However, in our study, as shown in Figure 6, the steep change in the 0.1 to 0.28 region poses some constraints on using this regression to predict %D/g from TUNEL positive cells, or vice versa.

Our study demonstrated that the early phase apoptosis induced by radiation is dose dependent, and ^99m^Tc-HYNIC-annexin V imaging can reflect this dose-response relationship. In EL4 lymphoma, the number of apoptotic cells detected by TUNEL in irradiated groups increased as radiation dose rose and was 1.7 to 4.9 times that of the un-irradiated groups. Within the same tumor tissue, the TUNEL results correlated well with the *in vivo *annexin V radioactivity which in the irradiated groups' uptake was also 1.7 to 4.9 times that in the un-irradiated tumors. Though we did not quantify the ^99m^Tc-HYNIC-annexin V uptake in TAVS, it could be visualized clearly that the intensity of tracer increased as the radiation dose escalated (Figures 2 and 3). Yong et al. [16] also reported similarly, on a murine breast tumor model, that it is feasible to use ^99m^Tc-EC-annexin to image early tumor apoptosis. Our results are consistent with a study reported by Liu [17]. However the positive correlation between early phase apoptosis and radiation dose is considered only applicable within a limited dose range [18]. Recent findings have been reported that large single dose irradiation (8 to 15 Gy) may enhance tumor radiation sensitivity through the induction of tumor blood vessel endothelium apoptosis [19,20].

Our study also illustrated that the degree of early phase apoptosis after irradiation might be correlated with tumor radiation sensitivity. When receiving the same irradiation dose, the EL4 lymphoma and S180 sarcoma responded differently. With a single 8 Gy irradiation, the EL4 tumor was completely controlled after radiation. This is consistent with the finding that El4 lymphoma is sensitive to radiation and usually undergoes P53 dependent apoptosis after radiation [21]. However, the S180 sarcoma was comparatively irradiation resistant as the tumor in this study remained stable for a short time after the same radiation dose and eventually relapsed. Meanwhile, after the same 8 Gy dose, the uptake of radio-labeled annexin V as well as the number of apoptotic cells detected by TUNEL in S180 sarcoma tumor was significantly lower than that in EL4 lymphoma, indicating that the different response to radiation between these two tumor types might be correlated with the difference in early phase apoptosis. Our study had a similar observation as that reported in the literature [7,8] that ^99m^Tc-HYNIC-annexin V accumulation correlated well with tumor response after radiotherapy in different tumor types. As this is a feasibility study, whether detection of apoptosis by ^99m^Tc-HYNIC-annexin V imaging might predict tumor radiation-sensitivity needs further validation.

In addition, the number of apoptotic cells at 0 Gy (without irradiation) was higher in EL4 tumor than in S180 sarcoma, indicating that the rate of spontaneous apoptosis in EL4 lymphoma is higher than that in S180 sarcoma. According to our results, the difference in spontaneous apoptosis was also positively correlated with the difference in degree of radiation-induced apoptosis. This suggested that pre-treatment spontaneous apoptosis might predict the apoptotic radiation response as well. Dubray also came to similar conclusions after studying the relationship between spontaneous and radiation-induced apoptosis with radiotherapy outcome in non-Hodgkin's lymphoma [22]. Rottey et al [23] utilized ^99m^Tc-HYNIC-annexin V imaging in head and neck squamous carcinoma to evaluate apoptosis before treatment, and found that spontaneous apoptosis in tumor could predict tumor response to treatment. Recently annexin V imaging has begun to be applied in patients' receiving head and neck tumor radiotherapy, but the significance is not clear and needs further investigation [24].

## Conclusion

Results of this preliminary study indicated that ^99m^Tc-HYNIC-annexin V imaging might provide a possible means of in vivo prediction of tumor response to radiation. The degree of early phase accumulation of ^99m^Tc HYNIC-rh-annexin V in tumor after single dose radiation implied radiation-induced apoptosis and radio-responsiveness. On the contrary, the tumor with no significant accumulation of ^99m^Tc HYNIC-rh-Annexin V implies poor response to radiotherapy.

## Competing interests

The authors report no conflicts of interest. The authors alone are responsible for the content and writing of the paper.

## Authors' contributions

GM-F and ZY-Q carried out the in vivo and in vitro studies, participated in drafting the manuscript. RT and LL participated in the In vivo imaging. GL-M carried out the establishment of tumor model. XF participated in designing and the execution of the experiment. YB-H and SB provided irradiation. WJ conceived and designed the study, helped analysing data and drafting the manuscript. All authors read and approved the final manuscript.
